# Genotype-environment interaction for grain yield in maize (*Zea mays* L.) using the additive main effects and multiplicative interaction (AMMI) model

**DOI:** 10.1007/s13353-024-00899-4

**Published:** 2024-08-08

**Authors:** Jan Bocianowski, Kamila Nowosad, Dariusz Rejek

**Affiliations:** 1 Department of Mathematical and Statistical Methods, Poznań University of Life Sciences, Wojska Polskiego 28, 60-637 Poznań, Poland; 2 Department of Genetics, Plant Breeding and Seed Production, Wrocław University of Environmental and Life Sciences, Wrocław, Poland; 3 Plant Breeding Smolice Co. Ltd, Kobylin, Poland

**Keywords:** Stability, Corn, Genotype selection index, Multi-locations experiment, Hybrids

## Abstract

Genotype-environment interaction consists of the different response of individual genotypes resulting from changing environmental conditions. Its significance is a phenomenon that makes the breeding process very difficult. On the one hand, the breeder expects stable genotypes, i.e., yielding similarly regardless of environmental conditions. On the other hand, selecting the best genotypes for each region is one of the key challenges for breeders and farmers. The aim of this study was to evaluate genotype-by-environment interaction for grain yield in new maize hybrids developed by Plant Breeding Smolice Co. Ltd., utilizing the additive main effects and multiplicative interaction (AMMI) model. The investigation involved 69 maize (*Zea mays* L.) hybrids, tested across five locations in a randomized complete block design with three replications. Grain yield varied from 8.76 t ha^–1^ (SMH_16417 in Smolice) to 16.89 t ha^–1^ (SMH_16043 in Płaczkowo), with a mean yield of 13.16 t ha^–1^. AMMI analysis identified significant effects of genotype, environment, and their interaction on grain yield. Analysis of variance indicated that 25.12% of the total variation in grain yield was due to environment factor, 35.20% to genotypic differences, and 21.18% to genotype by environmental interactions. Hybrids SMH_1706 and SMH_1707 are recommended for further breeding programs due to their high stability and superior average grain yield.

## Introduction

Environmental conditions can influence genotypes in various ways, leading to genotype by environment interactions (GEI) (Grishkevich and Yanai 2013; de Leon et al. 2016; Baldassarre et al. 2023; Gan et al. 2023; Oroian et al. 2023). While some genotypes exhibit consistent phenotypic traits across different environments, others demonstrate significant variability (Williams et al. 2008; Khare et al. 2024; Kumar et al. 2024). These interactions can cause substantial variation in yield performance among different genotypes under varying environmental conditions (Shrestha et al. 2012; Oladosu et al. 2017; Chauhan et al. 2023; Pour-Aboughadareh et al. 2023). GEI can be statistically defined as the discrepancy between the observed phenotypic performance and the predicted value from a model that considers the overall mean and main effects of genotype and environment (Bocianowski and Prażak 2022; Nowosad et al. 2023). Multi-environmental studies often reveal that traits such as yield and its components vary under different environmental conditions, classifying somo varieties as unstable (Vaezi et al. 2019; Ruswandi et al. 2023; Saeidnia et al. 2023). While major environmental effects can explain of this variation, GEI also contribute significantly to the differing performances of genotypes across environments. Breeders and farmers prefer varieties that exhibit stability or minimal environmental modification (Ceccarelli 1994; Atlin et al. 2017; von Gehren et al. 2023; Abdala et al. 2024). Assessing the stability of a genotype involves testing its general and specific adaptation through a series of trails (Ahakpaz Karkaji et al. 2023; Amelework et al. 2023; do Couto et al. 2023; Fekadu et al. 2023; Taleghani et al. 2023).

One primary goal in maize breeding is to consistently increase grain yield to enhance productivity (Duvick 2005). Understanding the genetic determinants of grain yield aids breeders in managing genetic improvements (Boote et al. 2001; Swarup et al. 2021). The most desirable varieties for agriculture are those with stable, high average yields or other essential traits, adapted widely to the target region (Ceccarelli 1994; Saeidnia et al. 2023; Kwambai et al. 2024). Varieties with narrow adaptation to specific habitat conditions are less frequently preferred.

Grain yield is a complex quantitative trait influenced by genotype, environmental factors, and GEIs. The differential response of genotypes to varying environmental conditions during plant growth adds to this complexity. GEI is often analyzed using the additive main effects and multiplicative interaction (AMMI) model (Mandel 1961, 1971; Gauch 1988; Zobel et al. 1988). The AMMI model, as proposed by Crossa (1990), combines the strengths of analysis of variance (ANOVA) and principal component analysis (PCA) through singular value decomposition (SVD). Initially, AMMI analysis estimates the main effects of genotypes and environments using ANOVA (Ferraudo and Perecin 2014; Hongyu et al. 2014; Demelash 2024; Patel et al. 2024).

The objective of this study was to evaluate genotype by environment interaction for grain yield in maize (*Zea mays* L.) grown in South Poland using the AMMI model.

## Material and methods

### Plant material

The study material comprised 69 maize (*Zea mays* L.) genotypes, including 66 experimental hybrids bred by Plant Breeding Smolice Ltd. IHAR Group and three cultivars (NK Ravello, Ricardinio, and ES Gallery). The 66 experimental hybrids are genotypes intended for use for grain, resulting from the crossbreeding of two opposite heterotic groups, which are forms with grain type dent and forms with grain type flint. These objects are characterized by intermediate flint/dent grain type. Thirty-one of them are single-cross varieties, and 35 objects are three-way cross varieties. The earliness of the hybrids tested ranged from about FAO 210 to about FAO 280. The final FAO value is indicated by Research Center for Cultivar Testing after 2-year registration experiments based on comparison with other reference varieties. The reference varieties are registered hybrids representing different earliness groups: NK Ravello, single-cross cultivar, grain type: flint, FAO number 210, intended for grain cultivation; Ricardinio, single-cross cultivar, grain type: flint/dent, FAO number 230/240, intended for grain and whole-crop silage cultivation; and ES Gallery, single-cross cultivar, grain type: dent, FAO number 270, intended for grain cultivation.

### Field experiment

**Fig. 1:**
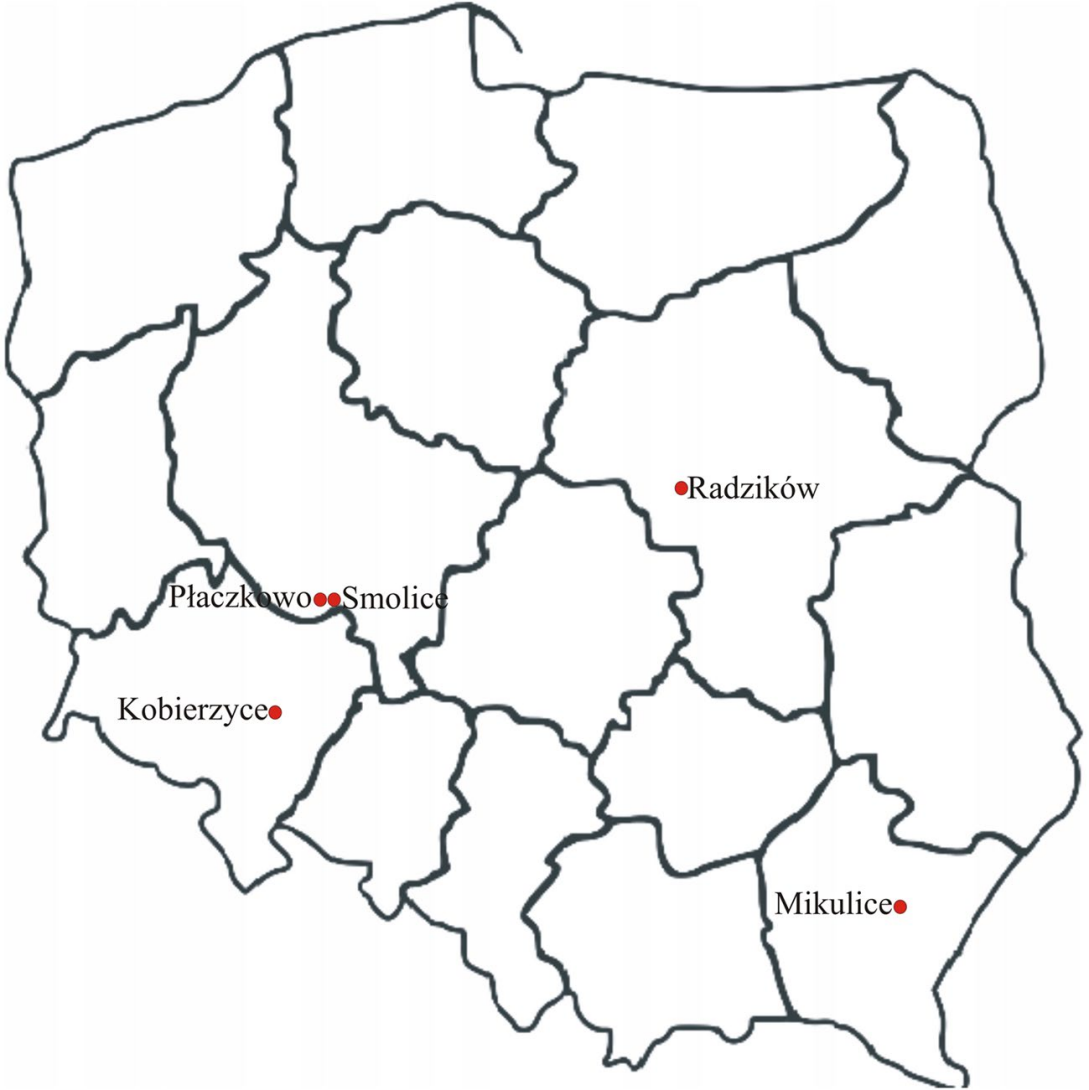
A map showing the location of the experiments

Field experiments were conducted across five locations with varying weather and soil conditions: Kobierzyce, Mikulice, Płaczkowo, Radzików and Smolice (Table 1, Fig. 1) in 2017. These experiments were arranged in a randomized complete block design with three replicates, on plots of 5.01 m^2^ (1.5 m × 3.34 m). Sowing was conducted using a precision drill in the second (E3, E5) or third decade of April (E1, E2, E4) and harvesting took place in the first (E2, E3) or second decade of October (E1, E4, E5). Agronomic practices were in accordance with accepted agricultural practice appropriate for each location. In accordance with this agricultural practice and knowledge of the field, winter plowing and spring tillage treatments appropriate to field conditions were applied. The following treatments were carried out: spear plowing, harrowing, and pre-sowing cultivation with tillage units. The following fertilization was applied in each locality: Kobierzyce, 125 kg ha^–1^ N, 50 kg ha^–1^ P, 120 kg ha^–1^ K; Mikulice, 139 kg ha^–1^ N, 80 kg ha^–1^ P, 120 kg ha^–1^ K; Płaszkowo, 160 kg ha^–1^ N, 94 kg ha^–1^ P, 170 kg ha^–1^ K, Radzików, 170 kg ha^–1^ N, 80 kg ha^–1^ P, 120 kg ha^–1^ K; Smolice, 145 kg ha^–1^ N, 110 kg ha^–1^ P, 160 kg ha^–1^ K. The preceding crops were maize (E1, E4, E5), winter rapeseed (E2), and winter wheat (E3). The experiments were conducted on class II (E3, E4), III a (E1, E2), and III b (E5) soils. Grain yield was standardized to 14% moisture content, and dry matter content was measured using the dryer method. In addition to the basic agronomic traits of yield and dry matter content at harvest, the following were also observed: number of plants infested with head blight (pcs), pre-harvest lodging (pcs), and cob flowering—the number of days from sowing to the appearance of nevi in 50% of the plants in the plot, plant height (cm) and height of setting of the first cob (cm).

**Table 1:** Geographic coordinates of the locations where the experiments were conducted, as well as total monthly rainfall and mean monthly air temperatures recorded there

Location	Kobierzyce	Mikulice	Płaczkowo	Radzików	Smolice
Environment symbol	E1	E2	E3	E4	E5
Latitude	50°58′19.411″N	50°00′35.964″N	51°41′13.102″N	52°13′444″N	51°42′12″N
Longitude	16°55′47.323″E	22°25′40.654″E	17°06′50.957″E	20°37′949″E	17°10′10″E
Total monthly rainfall [in mm]					
April	69.0	41.0	43.9	58.0	50.0
May	22.4	54.8	31.6	57.0	38.0
June	52.0	38.2	65.8	126.6	43.6
July	141.1	78.9	71.0	70.0	79.8
August	43.6	33.0	92.0	35.6	73.6
September	70.6	97.2	62.8	153.0	53.4
October	59.2	64.0	73.7	89.6	67.8
November	21.0	13.6	42.8	44.4	33.0
Mean monthly air temperature [in °C]					
April	8.5	8.8	7.6	7.6	8.1
May	14.9	14.1	14.1	14.6	14.3
June	19.4	18.9	18.4	18.4	18.4
July	19.7	19.3	18.9	19.0	18.8
August	20.1	20.4	19.4	19.6	19.7
September	13.5	14.3	13.1	14.0	13.8
October	11.4	10.3	10.5	10.0	11.1
November	6.3	4.7	5.3	5.1	5.8

### Statistical analysis

A two-way fixed effect model was applied to quantify the main effects of variability and their interaction on grain yield. Least-squares means were calculated using the AMMI model. Initially, the model fits additive effects for the main effects of genotypes (G) and environments (E), followed by multiplicative effects for GEI using principal component analysis (PCA). The AMMI model (Gauch and Zobel 1990) is formulated as

1 $${y}_{g,e,k}=\mu +{\alpha }_{g}+{\beta }_{e}+\sum_{n=1}^{N}{\lambda }_{n}{\gamma }_{g,n}{\delta }_{n,e}+{Q}_{g,e}+{\epsilon }_{g,e,k},$$

where *y*_*g,e,k*_ is the grain yield mean of genotype *g* in environment *e* for replicate *k*, *μ* is the grand mean, *α*_*g*_ is the genotypic mean deviation, *β*_*e*_ is the environmental mean deviation, *N* is the number of PCA axes retained in the adjusted model, *λ*_*n*_ is the eigenvalue of the PCA axis *n*, *γ*_*g,n*_ is the genotype score for PCA axis *n*, *δ*_*e,n*_ is the score eigenvector for PCA axis *n*, *Q*_*g,e*_ is the residual containing all multiplicative terms not included in the model, and *ϵ*_*g,e,k*_ is the experimental error. The expected distribution of *Q*_*g,e*_ is normal. The interaction matrix, post-residualization captures the GEI effects, assumed to be multiplicative and estimated via PCA. The approximate *F*-test by Gollob (1968) was used to determine the optimal number of multiplicative terms required within the AMMI model.

The AMMI stability value (ASV) was used to assess genotype stability (Purchase et al. 2000):

2 $$ASV=\sqrt{{\left[\frac{{SS}_{IPCA1}}{{SS}_{IPCA2}}\left({IPCA}_{1}\right)\right]}^{2}+{\left({IPCA}_{2}\right)}^{2}},$$

where *SS* is the sum of squares and IPCA1 and IPCA2 are the first and the second principal component axes of the interaction, respectively. The IPCA_1_ and IPCA_2_ scores are the genotypic scores in the AMMI model. A lower ASV indicates greater genotype stability across environments.

A genotype selection index (GSI) was calculated for each genotype, combining average grain yield and ASV into a single criterion (GSI_*i*_) as (Farshadfar and Sutka 2003):

3 $${\text{GSI}}_{\text{i}}={\text{RY}}_{\text{i}}+{\text{RASV}}_{\text{i}},$$

where GSI_*i*_ is the genotype selection index for *i*-th genotype, RY_i_ is the rank of the average grain yield for *i*-th genotype, and RASV_i_ is the rank of the AMMI stability value for the *i*-th genotype. All analyses were performed using GenStat v. 23.1 statistical software (VSN International 2023).

## Results

**Fig. 2:**
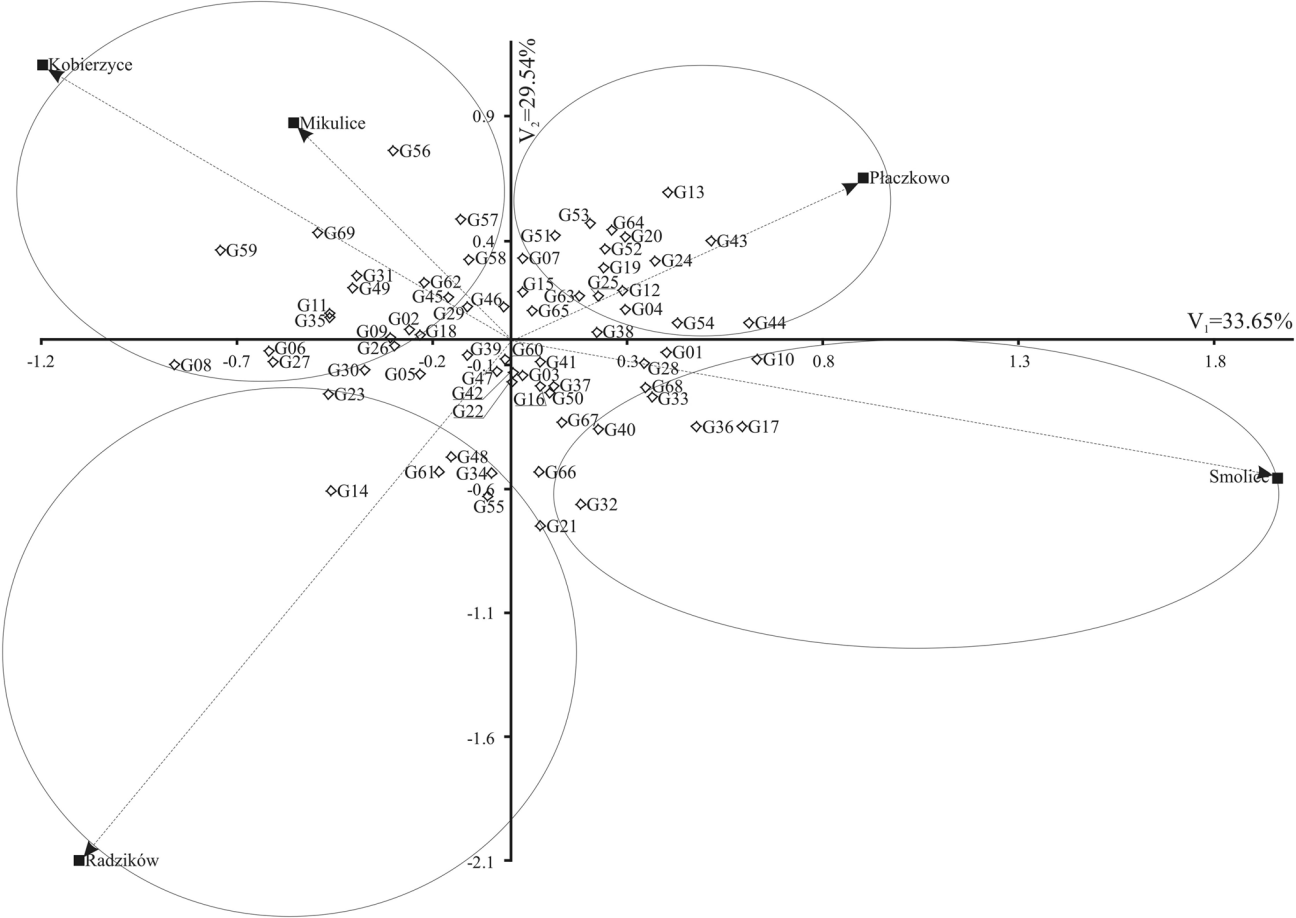
Biplot for genotype-environment interaction of maize (*Zea mays* L.) lines in six environments, showing the effects of the first and second components (IPCA1 and IPCA2, respectively)

The analysis of variance revealed that the sum of squares for the main effect of genotypes constituted 35.20% of the total variation, having the most significant impact on grain yield. Environmental differences accounted for 25.12% of the total grain yield variation, while GEI effects explained 21.18% (Table 2). These components were all highly significant. The first three principal components of GEI collectively explained 84.30% of the total variation in grain yield, with the first principal component (IPCA 1) accounting for 33.65%, IPCA 2 for 29.54%, and IPCA 3 for 21.11% (Fig. 2, Table 2).

**Table 2:** Analysis of variance of main effects and interactions for maize (*Zea mays* L.) genotypes grain yield

Source of variation	d.f	Sum of squares	Mean squares	*F*-statistics	Variability explained (%)
Genotypes (G)	68	853.4	12.55	19.71***	35.20
Environments (E)	4	609.1	152.26	98.91***	25.12
GE interactions	272	513.5	1.89	2.97***	21.18
IPCA 1	71	172.8	2.43	3.82***	33.65
IPCA 2	69	151.7	2.20	3.45***	29.54
IPCA 3	67	108.4	1.62	2.54***	21.11
Residual	65	80.6	1.24	1.95***	15.70
Error	680	432.9	0.64		

^***^p < 0.001. *IPCA* principal component of interaction, *d.f.* the number of degrees of freedom

Field test results highlighted the influence of weather conditions, environment, and genotypes on grain maize yield (Table 1). The grain yield of the tested genotypes varied from 8.76 t ha^–1^ (SMH_16417 in Smolice) to 16.89 t ha^–1^ (SMH_16043 in Płaczkowo) across five locations, averaging 13.16 t ha^–1^ (Table 3). Line SMH_1706 had the highest average yield (14.92 t ha^–1^), while line SMH_16417 had the lowest (10.87 t ha^–1^). Average grain yield per location ranged from 11.81 t ha^–1^ in Smolice to 14.01 t ha^–1^ in Płaczkowo.

**Table 3:** Average grain yield (t ha^–1^), for genotypes and environments, principal component analysis values of maize (*Zea mays* L.) lines tested, AMMI stability value (ASV), and genotype selection index (GSI)

Genotype	Code	Kobierzyce	Mikulice	Płaczkowo	Radzików	Smolice	Mean	IPCAg1	IPCAg2	ASV	GSI
SMH_1682	G01	12.97	12.99	13.79	13.00	12.78	13.11	0.399	− 0.053	0.458	75
SMH_16002	G02	13.97	13.72	13.71	13.82	11.54	13.35	− 0.257	0.040	0.296	52
SMH_16041	G03	14.5	14.23	15.03	14.93	13.15	14.37	0.032	− 0.144	0.148	12
SMH_16043	G04	14.12	14.85	16.89	14.69	13.13	14.73	0.294	0.119	0.356	29
SMH_16045	G05	13.69	13.84	14.21	14.46	11.65	13.57	− 0.228	− 0.139	0.294	42
SMH_16048	G06	14.67	14.69	15.32	15.52	11.06	14.25	− 0.617	− 0.045	0.705	68
SMH_16086	G07	11.53	11.78	12.98	11.06	9.41	11.35	0.034	0.329	0.331	89
SMH_16099	G08	15.25	13.52	15.05	15.63	10.34	13.96	− 0.858	− 0.100	0.982	84
SMH_16107	G09	15.53	13.1	15.57	14.98	11.93	14.22	− 0.305	0.009	0.348	35
SMH_16129	G10	12.06	12.67	15.12	13.00	12.41	13.05	0.630	− 0.081	0.722	103
SMH_16130	G11	14.95	14.15	14.2	14.45	11.60	13.87	− 0.460	0.102	0.534	64
SMH_16139	G12	13.64	12.33	14.21	12.54	12.11	12.97	0.287	0.197	0.382	75
SMH_16144	G13	13.19	12.5	15.07	11.50	11.35	12.72	0.402	0.592	0.748	112
SMH_16145	G14	13.57	12.68	13.50	15.39	11.20	13.27	− 0.457	− 0.606	0.799	101
SMH_16164	G15	13.35	12.59	12.96	12.29	11.45	12.53	0.032	0.193	0.196	62
SMH_16165	G16	10.50	12.42	13.01	12.39	10.00	11.66	0.113	− 0.186	0.226	76
SMH_16201	G17	11.68	11.16	14.57	13.05	11.87	12.47	0.590	-0.351	0.759	118
SMH_16247	G18	13.37	12.76	12.68	12.98	11.01	12.56	− 0.230	0.020	0.263	67
SMH_16264	G19	13.76	13.16	13.51	12.34	12.43	13.04	0.239	0.288	0.396	74
SMH_16265	G20	12.48	11.94	12.33	10.68	11.13	11.71	0.294	0.416	0.534	111
SMH_16266	G21	12.01	12.64	13.73	14.91	11.94	13.05	0.076	-0.749	0.754	106
SMH_16295	G22	14.47	13.69	14.56	14.69	12.96	14.07	0.006	-0.174	0.174	19
SMH_16309	G23	14.20	13.14	12.62	14.27	11.47	13.14	− 0.463	-0.222	0.572	90
SMH_16315	G24	13.03	11.97	13.13	11.40	11.81	12.27	0.370	0.315	0.526	101
SMH_16322	G25	15.69	12.55	14.79	13.60	13.52	14.03	0.228	0.176	0.313	32
SMH_16334	G26	15.23	13.91	15.29	15.10	12.22	14.35	− 0.294	-0.023	0.335	29
SMH_16337	G27	15.24	13.18	13.92	14.84	11.29	13.69	− 0.605	-0.088	0.695	77
SMH_16379	G28	11.85	11.88	13.13	12.25	11.43	12.11	0.342	-0.098	0.402	90
SMH_16380	G29	13.47	12.63	13.73	12.93	11.00	12.75	− 0.109	0.133	0.181	57
SMH_16411	G30	12.81	13.62	11.33	13.11	11.06	12.39	− 0.370	-0.125	0.439	88
SMH_16417	G31	11.77	11.78	11.04	10.99	8.76	10.87	− 0.392	0.259	0.516	111
SMH_16453	G32	13.16	13.59	14.41	15.48	13.32	13.99	0.180	-0.662	0.693	69
SMH_16461	G33	12.6	11.43	12.31	12.42	12.31	12.21	0.363	-0.233	0.474	95
SMH_16467	G34	11.35	11.19	11.69	12.97	10.49	11.54	− 0.048	-0.536	0.539	116
SMH_16481	G35	12.49	11.82	11.28	11.90	9.34	11.36	− 0.460	0.087	0.532	113
SMH_1701	G36	14.36	12.81	15.46	14.84	14.12	14.32	0.473	-0.350	0.643	62
SMH_1702	G37	13.52	12.44	13.84	13.72	12.08	13.12	0.078	-0.189	0.209	50
SMH_1703	G38	13.89	12.97	14.81	13.59	12.51	13.56	0.223	0.030	0.256	41
SMH_1704	G39	13.45	13.26	14.57	14.02	11.38	13.34	− 0.110	-0.064	0.140	40
SMH_1705	G40	14.26	13.06	14.42	14.74	13.59	14.01	0.224	-0.359	0.441	47
SMH_1706	G41	15.27	14.51	15.71	15.32	13.79	14.92	0.076	-0.089	0.124	2
SMH_1707	G42	14.73	14.27	14.65	14.87	13.35	14.38	0.007	-0.136	0.136	9
SMH_1708	G43	13.62	13.7	14.87	12.41	12.98	13.51	0.511	0.395	0.704	85
SMH_1709	G44	13.71	13.21	14.65	13.09	13.76	13.68	0.610	0.065	0.698	80
SMH_1709_A	G45	14.16	12.83	13.76	13.16	11.41	13.06	− 0.156	0.169	0.246	54
SMH_1710	G46	14.97	12.93	15.42	14.07	12.22	13.92	− 0.016	0.130	0.131	18
SMH_1711	G47	15.27	13.11	15.53	15.04	12.89	14.37	− 0.034	-0.127	0.133	10
SMH_1712	G48	13.24	12.71	14.63	15.05	11.5	13.43	− 0.149	-0.471	0.501	70
SMH_1713	G49	15.38	13.44	14.51	14.12	11.57	13.8	− 0.401	0.210	0.503	60
SMH_1714	G50	13.55	11.10	12.59	12.91	11.94	12.42	0.103	-0.216	0.246	69
SMH_1715	G51	14.28	13.23	15.57	13.03	11.74	13.57	0.115	0.417	0.437	56
SMH_1716	G52	13.88	12.77	14.16	12.27	12.08	13.03	0.244	0.367	0.460	82
SMH_1717	G53	12.33	11.97	14.49	11.38	10.1	12.05	0.204	0.470	0.525	104
SMH_1718	G54	13.27	13.72	15.12	13.44	12.9	13.69	0.427	0.068	0.491	60
SMH_1719	G55	13.06	12.47	12.80	14.62	12.28	13.05	− 0.057	-0.630	0.634	97
SMH_1720	G56	13.89	13.16	12.50	11.07	10.19	12.16	− 0.297	0.761	0.833	125
SMH_1721	G57	14.38	14.30	13.34	12.56	11.93	13.3	− 0.126	0.483	0.504	76
SMH_1722	G58	14.50	13.62	14.25	13.19	11.83	13.48	− 0.106	0.324	0.346	53
SMH_1723	G59	13.80	12.99	13.81	13.06	8.88	12.51	− 0.742	0.362	0.919	120
SMH_1724	G60	15.33	12.22	14.46	14.23	12.79	13.81	− 0.011	-0.081	0.082	87
SMH_1725	G61	12.80	10.83	12.01	13.56	10.94	12.03	− 0.182	-0.532	0.571	113
SMH_1726	G62	14.42	13.63	14.30	13.57	11.53	13.49	− 0.219	0.230	0.339	51
SMH_1727	G63	13.65	13.81	14.18	13.13	12.45	13.44	0.177	0.176	0.268	47
SMH_1728	G64	14.52	13.73	15.41	13.00	12.64	13.86	0.259	0.441	0.530	63
SMH_1729	G65	13.47	13.05	14.72	13.34	11.52	13.22	0.055	0.115	0.132	40
SMH_1730	G66	12.48	12.00	13.33	14.08	11.75	12.73	0.075	-0.532	0.538	98
NK Ravello	G67	11.71	11.14	11.81	12.36	10.99	11.6	0.133	-0.335	0.367	92
Ricardinio	G68	12.60	13.21	15.06	13.89	12.33	13.42	0.348	-0.192	0.441	65
ES Gallery	G69	16.81	15.05	15.15	14.73	12.6	14.87	− 0.492	0.432	0.707	62
Mean		13.66	12.95	14.01	13.49	11.81	13.18				
IPCA e[1]		− 1.197	− 0.555	0.897	− 1.101	1.957					
IPCA e[2]		1.110	0.878	0.655	− 2.092	− 0.550					

**Fig. 3:**
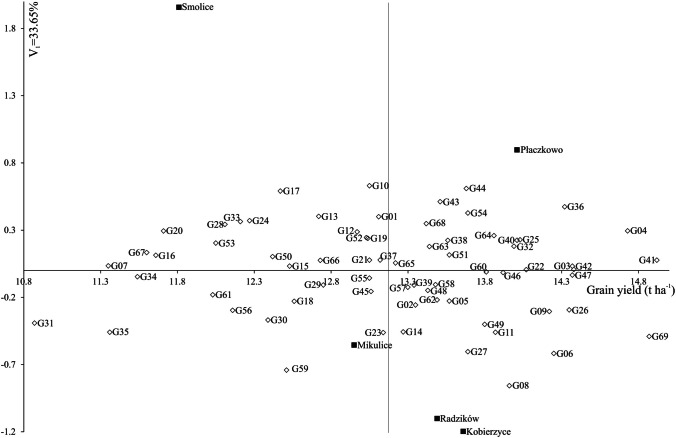
Biplot for the primary component of interaction (IPCA1) and average maize (*Zea mays* L.) grain yield (g). The vertical line in the center of the biplot is the overall grand mean

The AMMI1 biplot (Fig. 2) illustrates genotype and environment stability, along with specific GEIs. Among the genotypes, line SMH_16129 had the highest IPCA1 value of 0.630, whereas line SMH_16099 had the lowest IPCA1 value was –0.858 (Fig. 2, Table 2). Among the environments, Kobierzyce had the lowest IPCA1 value (–1.197), while Smolice had the highest (1.957) (Table 2, Fig. 2). Genotype stability, which indicates consistent performance across varying environmental conditions, was mainly influenced by climatic factors in this study. Figure 2 categorizes genotypes with specific adaptations into four groups. The stability of genotypes is further assessed by the biplot for grain yield (Fig. 3). Genotypes G59 (SMH_1723) and G69 (ES Gallery) interacted positively with Kobierzyce and Mikulice; line G14 (SMH_16145) with Radzików; G43 (SMH_1708) and G44 (SMH_1709) with Płaczkowo; and lines G01 (SMH_1682), G10 (SMH_16129), G17 (SMH_16201), and G33 (SMH_16461) with Smolice (Figs. 2 and 3). The analysis indicated that while some genotypes exhibit broad adaptation, most show specific adaptation. AMMI stability values (ASV) demonstrated variability in grain yield stability among the 69 genotypes (Table 3). A stable variety is characterized by an ASV value close to zero. Consequently, lines SMH_1706, SMH_1710, SMH_1729, SMH_1711, and SMH_1707, with ASV values of 0.124, 0.131, 0.132, 0.133, and 0.136 respectively, were the most stable. Conversely, lines such as SMH_16099 (0.982), SMH_1723 (0.919), SMH_1724 (0.919), and SMH_1720 (0.833) were the least stable (Table 3). Genotypes located at the highest point in specific sections of the graph performed best in environments within the same section (Fig. 3). On the biplot, lines SMH_16309 and SMH_1729 stood out with average grain yields of 13.14 and 13.22 t ha^–1^, respectively, close to the overall average of 13.18 t ha^–1^. Line SMH_1729 was particularly notable for its high stability. Lines SMH_1706, SMH_16043, and the cultivar ES Gallery exhibited the highest average grain yields but with different adaptations (Figs. 2 and 3): SMH_16043 showed specific adaptation to Płaczkowo, ES Gallery cultivar to Kobierzyce and Mikulice, and SMH_1706 demonstrated the greatest stability and the highest average grain yield (14.92 t ha^–1^). SMH_1706 also achieved the best genotype selection index of 2 (Table 3).

## Discussion

Effect of environmental conditions on biological and commercial traits of maize is very significant. The results of field trials demonstrated the impact of weather conditions (especially influence of monthly rainfall), environment, and genotypes on the grain yield of maize hybrids. In 2017, the spring season was notably cool, with substantial precipitation occurring during the latter half. Grain yield in maize (*Zea mays* L.) is influenced by multiple genes, resulting in varying genotype performance across different environments (Singamsetti et al. 2021; Alam et al. 2022; Kimutai et al. 2023; Ljubičić et al. 2023; Sabitha et al. 2024). Alam et al. (2022) showed that the phenotypic coefficient of variance had a higher value than the genotypic coefficient of variance, indicating the influence of environment on yield expression. In addition, they found that high heritability, combined with high genetic advance, indicated additive effects of genes. Kimutai et al. (2023) identified some QTLs for maize yield under nitrogen regimes. These QTLs have significant value for further validation and possible rapid introgression into maize populations using marker-assisted selection. In this study, three sources of variation were highly significant, consistent with findings from other researchers. For instance, Yue et al. (2022) analyzed 18 advanced maize genotypes and one check hybrid over 2 years at 37 locations across seven provinces. Nzuve et al. (2013) examined the interaction of 49 genotypes in three environments, while Signor et al. (2001) reviewed data from the French Association Générale des Producteurs de Maïs trial network, including 132 hybrids and 229 environments over 12 years. They conducted GEI analysis for datasets of 1 year, for 2 consecutive years, and for a dataset of 12 years. The results indicate that the magnitude of the GEI variance was equal to or greater than the genotypic variance (Signor et al. 2001). Ljubičić et al. (2023) studied four genotypes of different maturity classes and vegetation periods at one location over two growing seasons. Matongera et al. (2023) evaluated 24 inbred lines from various nutritional categories for GEI across stress and non-stress environments over 2 years at 11 sites in Zimbabwe. do Couto et al. (2023) analyzed 13 varieties in nine locations based on their widespread cultivation. Bocianowski et al. (2019c) investigated 32 maize genotypes (19 inbred lines and their 13 F1 hybrids) in four environments (2 years in two locations) for grain yield. Dehghani et al. (2009) and Adu et al. (2013) found non-significant GEI for grain yield, with Dehghani et al. (2009) studying 12 late maize hybrids in 11 sites over two growing seasons in Iran and Adu et al. (2013) analyzing 100 genotypes (98 single-cross maize hybrids, one local three-way hybrid, Akposoe, and an open-pollinated variety, Dodzi) in three environments. Adu et al. (2013) observed a lack of significant GEI for grain yield, which they interpret as consistent expression of this trait across all environments tested. Multi-location experiments often use the AMMI biplot to visualize the main effect of genotypes in different environments. The AMMI model is frequently employed in multi-species studies (Brancourt-Hulmel and Lecomte 2003; Pacheco et al. 2005; Balestre et al. 2009; Abakemal et al. 2016; Edwards 2016; Pires et al. 2018; Bocianowski and Liersch 2022; Mousavi et al. 2024).

It is a valuable tool for identifying GEI patterns and improving response estimate accuracy. It allows for grouping genotypes based on response characteristics and detecting trends across environments (Bocianowski et al. 2019a, 2019b, 2021; Patel et al. 2023; Yang et al. 2023; Pramanik et al. 2024; Singh et al. 2024).

Researchers can identify robust varieties with competitive yields in various environments using this approach, extracting more information from GEIs (Nowosad et al. 2018). In Southern Poland, grain yield expression is significantly influenced by genotype and environment main effects, as well as GEI. The main effect of the environment was primarily due to precipitation differences between June and July in the study year, while temperature had a smaller impact. These findings align with physiological processes in grain development, where water deficit can disrupt grain formation and reduce yield, as noted by Warzecha et al. (2010).

AMMI analyses can detect genotypes best suited to specific environmental conditions by estimating genotype interaction effects in each environment (Alizadeh et al. 2020; Zulfqar et al. 2021; Qasemi et al. 2022; Arinaitwe et al. 2023; Shojaei et al. 2023; Okla et al. 2023; Oghan et al. 2024). For grain yield, a significant GEI was demonstrated using AMMI analysis. Ranking of first four AMMI selections per environment selections per environment for grain yield is as follows: G69, G41, G49, G11 (in Kobierzyce); G69, G41, G04, G06 (in Mikulice); G04, G41, G06, G51 (in Płaczkowo); G08, G06, G32, G14 (in Radzików); and G36, G41, G44, G25 (in Smolice).

High genotype stability is related to the AMMI stability value, which evaluates the main effects of genotype, environment, and significant GEIs displayed on a GE biplot. The importance of these facts was pointed out in their studies, among others: Khan et al. (2021), Hailemariam Habtegebriel (2022), and Greveniotis et al. (2023). AMMI models effectively measure the importance of environments, genotypes, and their interactions, providing a value that indicates genotype stability across all environments, given grain yield (Sabaghnia et al. 2008; Mohammadi et al. 2018, 2023; Pour-Aboughadareh et al. 2022).

## Conclusions

This research demonstrates the use of the AMMI model to analyze the impact of genotype by environment interactions on maize grain yield. Sixty-nine maize hybrids were evaluated across five different environments. The analysis of variance revealed a highly significant influence of genotypes, environments, and their interactions on grain yield. The stability analysis identified line SMH_1729 as having high stability. Additionally, lines SMH_1706 and SMH_16043, along with the variety ES Gallery, exhibited the highest average grain yields but with varying environmental adaptations. Specifically, line SMH_16043 was best adapted to the conditions in Płaczkowo, while the ES Gallery variety was best suited to Kobierzyce and Mikulice. In contrast, line SMH_1706 demonstrated the greatest stability and highest average grain yield across environments. This line had the optimal genotype selection index and is recommended for further inclusion in breeding programs and production. AMMI analysis most often describes the response of genotypes to varying environmental conditions using a limited number of multiplicative components, usually based on the first two or three principal components for GEI effects. In the present study, the percentage of interaction variation explained by the first two and three principal components was 63.19% and 84.30%, respectively. This meant a loss of information of 36.81% and 15.70%, respectively.

## Data Availability

The datasets generated during and analyzed during the current study are available from the corresponding author on reasonable request.
